# Emerging markets equities’ response to geopolitical risk: Time-frequency evidence from the Russian-Ukrainian conflict era

**DOI:** 10.1016/j.heliyon.2023.e13319

**Published:** 2023-01-30

**Authors:** Samuel Kwaku Agyei

**Affiliations:** Department of Finance, School of Business, University of Cape Coast, Cape Coast, Ghana

**Keywords:** Black swan, Emerging markets equities, Geopolitical risk, Russian-Ukrainian conflict, Time-frequency analysis, Diversification, Hedge, Safe-haven

## Abstract

This study investigates the asymmetric interdependence between geopolitical risk (GPR) and the stock markets of the top-seven emerging (E7) countries (i.e., Mexico, Russia, Turkey, India, China, Indonesia, and Brazil) in the ongoing geopolitical conflict between Russia and Ukraine. With daily datasets covering the period 01-Feb-2022 to 25-July-2022, the squared wavelet coherence (SWC) and wavelet coherence phase difference (WCPD) techniques are employed. The results underscore heterogeneous and asymmetric market-specific coherence and lead-lag patterns regarding E7 stocks' interdependence with geopolitical risk. The findings imply high comovements between Black Swan events like the Russian-Ukrainian conflict and financial markets' volatility, highlighting the essence of alternative assets or asset classes for hedging geopolitical risks in the ongoing military actions. The heterogeneous and asymmetric responses offered by E7 stocks against GPR render emerging markets equities suitable for diversification and downside hedging strategies against GPR-induced shocks. The findings are robust to the time-varying parameter vector autoregression (TVP-VAR) connectedness approach. The results’ implications for portfolio managers, investors, and policymakers are discussed.

## 1 Introduction

Emerging markets equities (EMEs) are an asset class that appeals to investors due to the important role they play in international portfolio diversification. EMEs yield high risk-adjusted returns on average and have evolving connections with equities from developed markets, making them desirable surrogates for constructing diversified portfolios [1,2].

Financial markets occasionally experience crises (popularly termed as Black Swan events^1^) that cause a change to the risk-reduction role of some assets and asset classes, causing portfolio rebalancing due to fluctuating correlations across investment horizons and bullish and bearish conditions of the market [3,4]. Therefore, to facilitate effective and timely asset allocation decisions during crises, knowledge of how the principal emerging markets respond to market shocks is crucial for international market participants such as investors and policymakers [5,6]. One significant event in the history of financial markets is Russia's invasion of Ukraine on 24-Feb-22. The ongoing military actions between Russia and Ukraine have increased geopolitical risk and tensions that have already affected the correlations between different assets [7].

Motivated by this significant market event, this study investigates the hedging potential of emerging markets against geopolitical risk. The ongoing Russian-Ukrainian military conflict has introduced stressed conditions whose impact is seen in several economic sectors [8,9]. The academic literature classifies military conflicts as a Black Swan event that impacts global equity markets through several channels. First, investors' uncertainty about the future profitability of firms is heightened by military conflicts and this results in rampant volatility in stock prices [10]. Second, at the expense of other economic sectors, defence expenditure increases during military conflicts. Third, existing trading connections between non-war and war economies are distorted during military conflicts [11], constraining firms’ production, profitability, projected cash flows, and share values. Thus, the impact of military conflicts on equity markets is critical to several financial market players and makes this study timely.

It is paramount to note that some of today's emerging markets will be tomorrow's developed markets [12]. To financial market participants, the major consequence resulting from this may be envisaged from the context of portfolio management. Developed markets are noted for high integration and, as such, assets from emerging markets appeal to international investors for hedging extreme risks during financial market meltdowns [13]. Hence, the need to test EMEs' resilience against geopolitical risk is aroused by the market shocks pioneered by the Russian-Ukrainian geopolitical conflict.

Inter alia, as the conflict persists, three main issues that are essential to be empirically addressed, for the sake of timely rebalancing of portfolios and market regulation, include: (i) whether geopolitical risk drives emerging markets’ equities; (ii) whether all EMEs offer equal responses to geopolitical risk; and (iii) whether the relationship between geopolitical risk and EMEs differ across time and frequencies.

For these aims, the study focuses on the top-seven emerging markets (i.e., E7, including Mexico, Russia, Turkey, India, China, Indonesia, and Brazil), which are most vulnerable to market shocks [14]. It is worth noting that the BRIC economies, which are noted to be the most significant emerging markets [1] are contained in the E7 markets. Hence, the study sample is representative of the most important emerging markets in the world.

The study's contribution to the body of knowledge is threefold. First, based on the existing knowledge, this is the first study to examine the risk reduction role of emerging markets against shocks from geopolitical risk in the Russian-Ukrainian conflict era. Second, unlike the existing works (as highlighted in the literature review) that primarily focus on the time domain, this study takes on analysis across both time and frequency spectrums. The need for time-frequency analysis is justified as follows. In the spirit of the fractal market hypothesis, as espoused by Peters [15], market participants' behaviour is unevenly spread across trading horizons. So, as markets respond rapidly to evolving events, investors also modify their risk-return preferences to suit the prevailing condition [16]. Resultantly, market dynamics are likely to be heterogeneous across trading horizons. It is worth noting that in times of Black Swan events, investors are tempted to engage in short-selling behaviour [9] even if they hold no special assets in short stocks. To account for these factors, it is important to employ econometric approaches that cater for the heterogeneous and complex character of the market. Based on the above argument, several studies employ the wavelet econometric technique due to its ability to deal with complex data structures without making assumptions about the statistical properties of the data. For instance, Umar et al. [17] use this approach to analyse the impact of COVID-19 on commodity markets using daily data that spans between February and July 2020. Similarly, Umar, Bossman, Choi and Vo [9] analyse the impact of the Russia-Ukraine geopolitical risk on short stocks from various sectors of economic activity by employing daily data between February and July 2022.

Third, in terms of methods, robust and advanced econometric approaches, the squared wavelet coherence (SWC) and the wavelet coherence phase difference (WCPD) under the bi-wavelet framework are employed. The SWC and WCPD allow the assessment of the degree of coherence and the lead-lag dynamics between bi-variate time series across calendar time and frequency domains, which other approaches lack. Providing time-frequency evidence of the impact of geopolitical risk on assets is particularly important in Black Swan period for which the era of the Russian-Ukrainian military conflict is no exception. Time-frequency evidence is necessary to inform the timely rebalancing of portfolios across time and trading horizons. In the context of this study, the use of the time-frequency analysis also helps to ascertain whether or not stock market dynamics are driven by geopolitical risk. Such knowledge is instrumental for the effective regulation of markets in periods of elevated geopolitical risk. Note that when other econometric techniques, such as spillover connectedness (e.g. Refs. [18,19]) and traditional regression approaches, are employed, we cannot observe the dynamics between GPR and various E7 stocks across calendar time and investment horizons simultaneously. Besides, the dynamics of lead-lag comovements cannot be portrayed by such methods. Therefore, the application of the SWC and WCPD techniques, which incorporate all the above-mentioned features, is adequate.

This study underscores medium-to-high time-frequency coherence between stock returns from E7 countries and the Russian-Ukrainian conflict-induced geopolitical risk (GPR) shocks. The study documents predominantly high comovements between GPR and E7 stocks, implying that there exists high coherence between Black Swan events like the Russian-Ukrainian conflict and financial markets’ volatility. Among the E7 markets, Russian stocks seem to have been more susceptible to GPR, mainly in the early weeks across the weekly to the monthly frequency band only whereas Turkish stocks possess high diversification potential.

The rest of the study is outlined as follows. Section 2 reviews related literature; Section 3 presents the data and methodology; Section 4 discusses the findings; Section 5 ascertains the robustness of the findings, and Section 5 concludes the study.

## 2 Literature review

The academic attention towards examining the extreme financial effects of Black Swan events has risen in recent periods because of the COVID-19 pandemic crisis, where various financial markets’ reactions and overall economic recovery have been investigated widely (see, e.g. Refs. [4,[20], [21], [22], [23], [24], [25]]). However, discussions on the impact of geopolitical and military actions on financial markets are relatively scanty. Three main strands of work are reviewed to lay the premise for this study.

The first strand covers earlier works that use terror and wars to proxy geopolitical risk. Among them, Frey and Kucher [26,27] document that wars negatively affect the prices of government bonds from war countries. Choudhry [28] reports that war events over the period 1939–1945 caused significant structural breaks in the returns and volatility patterns for US stocks while Hudson and Urquhart [29] document a weak impact of war events and UK stocks.

The second strand of works commenced when Caldara and Iacoviello [30] developed the new geopolitical risk index (GPR) based on computerised text searches. This index has been used to examine the impact of geopolitical risk on various asset classes, both conventional and unconventional (see, e.g. Refs. [[31], [32], [33], [34], [35]], and the references therein). Among regional stock markets, Choi [36] notes significant and high power comovements between GPR and the volatilities of North-East Asian stocks. In a cross-section of 19 emerging markets, Zaremba et al. [37] investigate the predictability of changes in GPR on stock returns. They report that future returns from EMEs are significantly predicted by the previous month's change in GPR.

The discussions on GPR's impact on various assets have been rekindled in the era of the Russian-Ukrainian conflict, occasioning the third strand of works. Thus, this strand of literature assesses the impact of the geopolitical shocks occasioned by the Russian-Ukrainian military actions on financial markets. This has seen discussions on currencies [38], asset types [39], economic sectors [8], and regional and world equities [[40], [41], [42]]. Common to these studies, GPR heterogeneously affects asset prices, returns, and volatilities.

From the third strand of works, which is still an emerging strand, three major issues are worth noting. First, the focus of these studies has largely been on global or developed markets. This study extends this emerging literature by focusing specifically on emerging markets equities to examine their potential to hedge against the geopolitical shocks brought about by Russia's evasion of Ukraine. Second, notable assessments of GPR's impact on stock markets by Yousaf et al. [41] and Sun et al. [42] were based on the event study approach and, hence, failed to reveal any evidence across the frequency domain. Boungou and Yatié's [40] work was based on a panel methodology, which conceals any market- or country-specific dynamics between GPR and stock markets. Besides, the contribution of Umar, Bossman et al. [9] focused on Russian and European assets and selected global commodities while that of Bedowska-Sojka et al. [8] focused on economic sectors. Thus, a study that captures specific emerging markets and also provides evidence from the time-frequency domain is non-existent, despite their ability to serve as desirable surrogates for portfolio diversification.

Third, between GPR and EMEs, the lead-lag dynamics, which are relevant to assessing the driver and responder of market shocks across the spectrums of time and frequencies (trading horizons), are unknown in the era of the Russian-Ukrainian geopolitical conflict. This Black Swan event stands the chance of causing changes to the dynamics of price, returns, and volatility in equity markets. As a result, the need for a timely rebalancing of portfolios, which is key in crisis periods, is strongly motivated and warrants that empirical assessments focus on how EMEs may behave across time and frequency scales.

Therefore, in the persistence of the military conflict between Russia and Ukraine, this study extends the literature by testing the ability of EMEs to hedge against geopolitical risk. Findings from this study should influence portfolio rebalancing and effective market regulation on the part of investors and policymakers or regulators, respectively. This study fills the existing gap by employing the wavelet coherence analysis to provide evidence of the lead-lag dynamics between geopolitical risk (GPR) and EMEs across the time-frequency space. The analysis in this study would help to also ascertain whether EMEs offer an equal response to GPR and whether the relationship between GPR and EMEs differs across time and trading horizons.

## 3 Materials and methods

### 3.1 Data description and sources

**Fig. 1:**
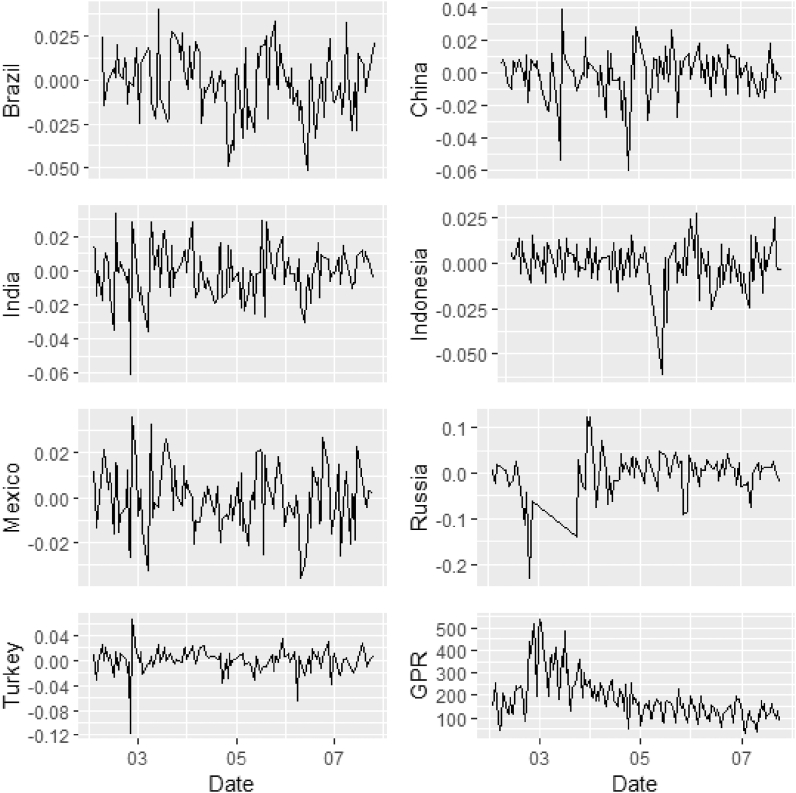
Plots of GPR index and E7 stock returns. *Notes:* This figure presents plots of the geopolitical risk index (GPR) and stock returns of emerging seven countries namely Brazil, China, India, Indonesia, Mexico, Russia, and Turkey and the daily geopolitical risk index (GPR). The horizontal (vertical) axis shows months (returns or index). The sample period starts from 01-Feb-2022 to 25-Jul-2022.

This study investigates the time-frequency-varying comovements between geopolitical risk (GPR) and the stock markets of the top-seven emerging (E7) countries. The daily GPR index developed by Caldara and Iacoviello [30] is employed in addition to the daily stock market returns of each of the E7 countries sourced from EquityRT. The sample period covers February 1, 2022 to July 25, 2022. The statistical properties of the data are detailed in Table 1 and the graphical trends of each data series are shown in Fig. 1.

**Table 1:** Sample statistics for GPR index and E7 stock returns.

	Brazil	China	India	Indonesia	Mexico	Russia	Turkey	GPR
Min	−0.0514	−0.0602	−0.0607	−0.0612	−0.0358	−0.2312	−0.1171	30.21
Max	0.04	0.0388	0.0332	0.0273	0.036	0.1223	0.0668	539.58
Median	−0.0008	0.0002	−0.0013	0.0016	−0.0018	0.006	0.0017	162.22
Mean	−0.001	−0.001	−0.0009	−0.0001	−0.0005	−0.0023	−0.0004	186.3044
Std. Dev	0.0178	0.0143	0.0147	0.012	0.0138	0.0468	0.0199	99.8249
Skewness	−0.305	−0.9561	−0.4766	−1.2338	0.0666	−1.2367	−1.7288	1.354
Kurtosis	−0.1013	3.0008	1.5106	4.9203	−0.1576	5.6484	10.0042	1.8252

*Notes:* This table presents the statistical properties of the sample. The sample includes the geopolitical risk index (GPR) and returns on emerging seven (E7) stock markets namely Brazil, China, India, Indonesia, Mexico, Russia, and Turkey. The sample period starts from 01-Feb-2022 to 25-Jul-2022.

The descriptive summary indicates that all E7 stock markets recorded negative returns on average, with Russia recording the most negative. Except for Mexico, all E7 market returns were negatively skewed over the sample period GPR peaked at 539.58 but had a minimum of 30.21. The trajectories of the return series for each of the E7 markets are suggestive of the volatile nature of EMEs across the sample period.

### 3.2 Methodology

This study makes use of the squared wavelet coherence (SWC) and wavelet coherence phase difference (WCPD) techniques, which are under the bi-wavelet paradigm. The methodological steps used by the study under the bi-wavelet approach are consistent with the basic steps of Torrence and Compo [43] and Torrence and Webster [44]. This approach has been propagated in recent literature [[45], [46], [47]]. The approach emanates from a bivariate framework based on a continuous wavelet transform (CWT) that does well in revealing different localized scales [48].

In generating the CWT of two distinct time series, say x(t) and y(t), we follow Torrence and Compo [43] to generate the SWC between x(t) and y(t) from their various CWTs, $W_{n}^{x}\left(u,s\right)$ and $W_{n}^{y}\left(u,s\right)$, respectively, in the presence of location u, sale s, and a complex conjugate × as in equation (1):

(1) $$W_{n}^{xy}\left(u,s\right)=W_{n}^{x}{\left(u,s\right)}^{*}W_{n}^{y}\left(u,s\right)\text{.}$$

The CWT permits a differentiation of the portions across time and frequencies, embodied by the coherence between x(t) and y(t), with or without mutual strong power. In other words, at each wavelet scale, the CWT portrays the localized covariance between x(t) and y(t). Hence, a CWT estimate near 1 implies that the two variables are highly synchronised and the opposite is true for an estimate near 0.

The SWC, which explains the comovements between x(t) and y(t) is defined by Torrence and Webster [44] in equation (2) as:

(2) $$R^{2}\left(u,s\right)=\frac{|S{\left(s^{-1}W^{xy}\left(u,s\right)\right)|}^{2}}{S\left(s^{-1}|W^{x}(u,s{)|}^{2}\right)S(s^{-1}|W^{y}(u,s{)|}^{2})}\text{,}$$

where S denotes smoothing on the time-frequency scale. The SWC parameters can be interpreted as a correlation measure in time-frequency space, with a range of values confined between 0 and 1. However, converse to the classical correlation measure between two sets of data arrays (i.e., Pearson coefficient, which ranges between −1 and 1), the SWC by default, falls within the interval 0 to 1. Thus, it fails to detect whether the found coherence lies in the same or opposing paths. Put differently, in classifying negative and positive coherences, reliance cannot be placed on the SWC.

To gain further insights into the lead-lad dynamics and the coherence patterns between x(t) and y(t), the WCPD comes to play. The WCPD facilitates the distinction between two ostensible relations, i.e., positive and negative [43].

The WCPD can be expressed in equation (3) as:

(3) $$\Phi_{ab}\left(u,s\right)=\tan^{-1}\left(\frac{Im\{S\left(s^{-1}W^{xy}\left(u,s\right)\right)\}}{Re\{S\left(s^{-1}W^{xy}\left(u,s\right)\right)\}}\right)\text{,}$$

where Im (Re) denotes imaginary (real) segments of the joint smoothed CWT.

A set of two data arrays with a null phase difference is an example of a perfectly co-moving time series. A standard visual representation of the data based on heat map panels is adopted to represent both SWC and WCPD. In the SWC heat maps, deep arrows reflect phase connections between any named two series (GPR and any of E7 equities).

The data arrays act in either in-phase (positive correlation) or anti-phase (negative correlation) mode, portrayed by the left- and right-oriented arrows, respectively. Arrows pointing upward or downward signify that x(t) and y(t) is ahead of y(t) and x(t), respectively by π/2. Taking note of the guidelines spelt out above facilitates deciphering the message covered by an arrow, regardless of the direction it points.

## 4 Results

This section presents the empirical results from the SWC and WCPD-based lead-lag relationships between GPR and each of the E7 stock markets. The patterns of comovement from which the lead-lag dynamics could be inferred are presented in scalograms. All horizontal axes display the time and the vertical axes denote the frequency, measured in days. The colour map accompanying each scalogram represents a key for defining the degree of comovement. Hotter (warmer) colours, shown by red and yellow (blue and green), show high (low) coherence between the pair. Graphically, → and ← oriented arrows suggest in-phase and out-phase coherence, respectively. Right and up (↗) or left and down (↙) arrows evidence a leading role for the first variable (GPR). Right and down (↘) or left and up (↖) arrows indicate a leading role for the second variable (the named E7 stock). Emphasis is laid on the phase difference relationships that fall within the cone of influence (COI), which depicts the significant comovement dynamics between the analysed pairs. The COI shows on the scalogram as the faded area. That is, the lead-lag dynamics in (outside) the clear regions are significant (non-significant).

### 4.1 Geopolitical risk and brazilian stocks

**Fig. 2:**
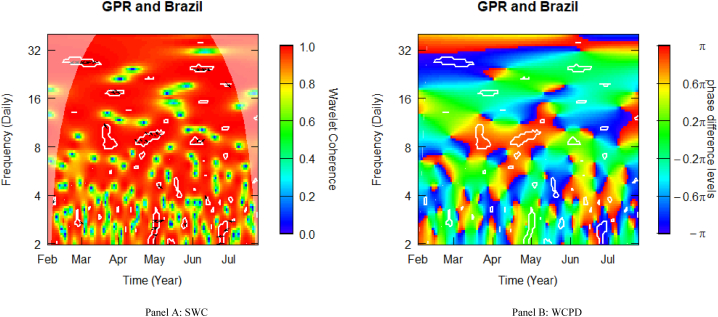
Wavelet analysis: geopolitical risk (GPR) index and stock returns in Brazil. *Notes:* This figure shows the squared wavelet coherence (left plot, i.e., Panel A) and wavelet coherence phase difference (right plot i.e., Panel B) between GPR and stock returns in Brazil. The horizontal (vertical) axis shows time steps in months (frequency scales in days). The sample period starts from 01-Feb-2022 to 25-Jul-2022. White contours depict significant reactions and are interpreted based on the arrows. ← and → arrows show in-phase and anti-phase comovements; ↗ or ↙ arrows indicate a lead role for the first variable (GPR); ↘ or ↖ indicates a lead role of the second variable (stock returns). GPR leads (lags) stock returns by π/2 with ↑ (↓) arrows. The colour bar shows the strength of comovements – hotter (yellow to red) colours signify strong comovements and colder colours (green to blue) signify weak comovements.

Fig. 2 depicts the SWC measure and the WCPD-based lead-lag dynamics between GPR and stock returns from Brazil. For this pair (GPR-Brazil), this study documents varying coherence levels of high, medium, and low. It is paramount to note that the heatmap is mostly red above the fortnightly scale (i.e., above 16 daily cycles), depicting a high coherence during the sample period analysed. This is especially observable in the left upper quadrant, suggesting that during Russia's invasion of Ukraine in February 2022, stock markets underwent stress conditions. The consistent high coherence is evidence of the high comovements between the geopolitical risk (GPR) shocks propagated by the Russian-Ukrainian conflict and stock market returns from Brazil. The cloud of left-downward-oriented positioning arrows observed in this time-frequency region is an indication of a negative correlation, which is confirmed by the bright blue colour surrounded by the white contour in the WCPD plot (see Fig. 2-panel B). This indicates that returns on Brazilian stocks were negatively driven by GPR from the onset of Russia's invasion of Ukraine. This observation is coherent, as EMEs are highly volatile during market crises [49]. Thus, investors would rather observe or get insights into the next step from Russia's invasion before balancing portfolios. This explains why Brazilian equities were rather driven by GPR across such periods. This observation stresses that the wavelet coherence analysis gives a correct reflection of the underlying reality of the ongoing military action.

At high frequencies (2–4 daily periodicities) towards the end (beginning) of April (May), GPR led Brazilian stocks, which is consistent with what was observed from the onset of the conflict. After 2 months into the conflict, Brazilian stocks were now negatively driving GPR, particularly towards the end of June. The lead-lag dynamics across the weekly and fortnightly (8–16) frequency bands across the period were characterised by high coherence, as depicted by red and green colours from the WCPD plot (see Fig. 2-panel B), casting doubts about the hedging potential of Brazilian stocks. A similar observation could be made from the monthly scale corresponding to June.

### 4.2 Geopolitical risk and Chinese stocks

**Fig. 3:**
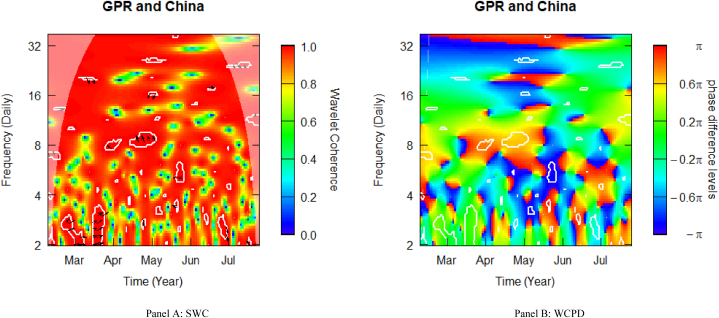
Wavelet analysis: geopolitical risk (GPR) index and stock returns in China. *Notes:* This figure shows the squared wavelet coherence (left plot, i.e., Panel A) and wavelet coherence phase difference (right plot, i.e., Panel B) between GPR and stock returns in China. The horizontal (vertical) axis shows time steps in months (frequency scales in days). The sample period starts from 01-Feb-2022 to 25-Jul-2022. White contours depict significant reactions and are interpreted based on the arrows. ← and → arrows show in-phase and anti-phase comovements; ↗ or ↙ arrows indicate a lead role for the first variable (GPR); ↘ or ↖ indicates a lead role of the second variable (stock returns). GPR leads (lags) stock returns by π/2 with ↑ (↓) arrows. The colour bar shows the strength of comovements – hotter (yellow to red) colours signify strong comovements and colder colours (green to blue) signify weak comovements.

Fig. 3 depicts the SWC measure and the WCPD-based lead-lag dynamics between GPR and stock returns from China. For the GPR-China pair, this study documents varying coherence levels of high, medium, and low. Similar to what was observed in the case of Brazil, the heatmap is mostly red above the fortnightly scale (i.e., above 16 daily cycles), depicting a high coherence during the sample period analysed. This is especially observable in the left upper quadrant, suggesting that during Russia's invasion of Ukraine in February 2022, Chinese stock markets underwent stress conditions. The consistent high coherence is evidence of the high comovements between the geopolitical risk (GPR) shocks propagated by the Russian-Ukrainian conflict and stock market returns from China. The cloud of right-upward-oriented arrows observed in this time-frequency region, particularly across the high-frequency (2–4 daily) band is an indication of a mild positive correlation, which is confirmed by the green colour surrounded by the white contour in the WCPD plot (see Fig. 3-panel B). This indicates that returns on Chinese stocks were positively driven by GPR shocks from the onset of Russia's invasion of Ukraine. This observation confirms the highly volatile character of EMEs during crises. Thus, GPR shocks emerge before stock market dynamics in China.

Towards the end of May (around 5 daily cycles) and June (around 2–3 daily cycles), the scalogram shows left-oriented arrows, suggesting a negative comovement between GPR shocks and stock returns from China and highlighting potential diversification with Chinese stocks. This observation is confirmed by the white contours coloured in blue and turquoise in May and June, respectively (see Fig. 3-panel B). It may be noted that this observation is comprehensible since, after some time into the conflict, market participants might have been saturated with GPR shock spillovers. Across the time-frequency space, this diversification seems to have also manifested at the fortnight scale towards the end (spring) of May (June). However, this does not last as it is overturned in mid-June at the same scale. The implication is that stock market dynamics in China largely responded to GPR shocks as they evolve.

### 4.3 Geopolitical risk and indian stocks

**Fig. 4:**
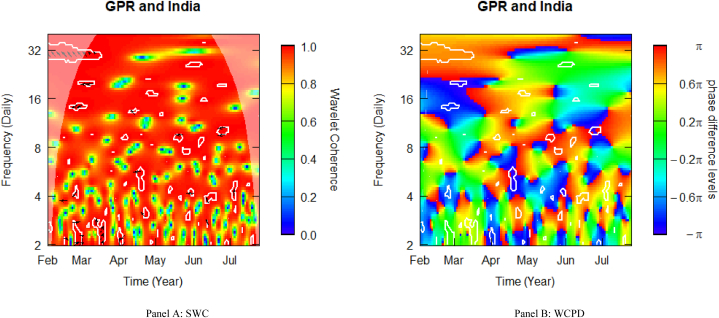
Wavelet analysis: geopolitical risk (GPR) index and stock returns in India. *Notes:* This figure shows the squared wavelet coherence (left plot, i.e., Panel A) and wavelet coherence phase difference (right plot, i.e., Panel B) between GPR and stock returns in India. The horizontal (vertical) axis shows time steps in months (frequency scales in days). The sample period starts from 01-Feb-2022 to 25-Jul-2022. White contours depict significant reactions and are interpreted based on the arrows. ← and → arrows show in-phase and anti-phase comovements; ↗ or ↙ arrows indicate a lead role for the first variable (GPR); ↘ or ↖ indicates a lead role of the second variable (stock returns). GPR leads (lags) stock returns by π/2 with ↑ (↓) arrows. The colour bar shows the strength of comovements – hotter (yellow to red) colours signify strong comovements and colder colours (green to blue) signify weak comovements.

Fig. 4 depicts the SWC measure and the WCPD-based lead-lag dynamics between GPR and stock returns from India. Similar to other pairs, this study documents varying coherence levels of high, medium, and low for the GPR-India pair. As was observed previously, the heatmap is mostly red above the fortnightly scale (i.e., above 16 daily cycles), depicting a high coherence during the sample period analysed. This is especially observable in the left upper quadrant, corroborating the spring of the Russia-Ukraine military actions and suggesting that during Russia's invasion of Ukraine in February 2022, Indian stock markets underwent stress conditions. The consistent high coherence confirms the high comovements between the geopolitical risk (GPR) shocks propagated by the Russian-Ukrainian conflict and stock market returns from India in the study period. The cloud of right-upward- and downward-oriented arrows observed in this time-frequency region, particularly across the high frequency (2–4 daily) band between February and late April is an indication of a mild positive coherence, which is confirmed by the green colour surrounded by the white contour in the WCPD plot (see Fig. 4-panel B). This indicates that returns on Indian stocks were positively driven by GPR shocks from the onset of Russia's invasion of Ukraine. As previously mentioned, this observation confirms the highly volatile character of EMEs during crises.

It is worth noting that Indian stocks signalled some diversification potentials across 4–6 and 12–24 daily cycles in late (early) March (April). This lends support to the fact that equities from emerging markets tend to yield high risk-adjusted returns on average and have evolving connections with equities from developed markets, rendering them attractive surrogates for diversifying portfolios [1,2]. However, given the strength of the Black Swan (i.e., the military conflict between Russia and Ukraine) period, this diversification potential was overturned at lower frequencies, particularly around 28–32 daily periodicities. This is further confirmed by the intermittent positioning arrows found between April and July across the time-frequency spectrum, failing to indicate any consistent lead-lag pattern. This is attributable to the high uncertainty left with market participants amid the military conflict.

### 4.4 Geopolitical risk and Indonesian stocks

**Fig. 5:**
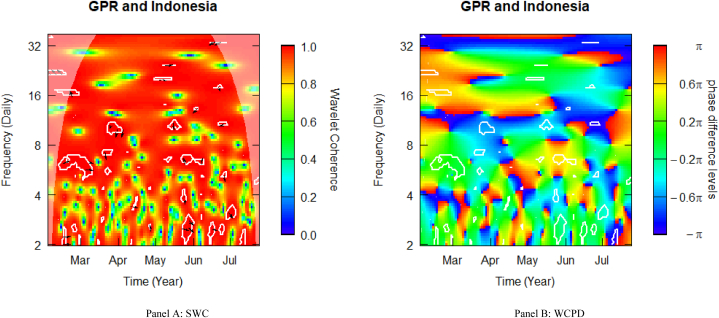
Wavelet analysis: geopolitical risk (GPR) index and stock returns in Indonesia. *Notes:* This figure shows the squared wavelet coherence (left plot, i.e., Panel A) and wavelet coherence phase difference (right plot, i.e., Panel B) between GPR and stock returns in Indonesia. The horizontal (vertical) axis shows time steps in months (frequency scales in days). The sample period starts from 01-Feb-2022 to 25-Jul-2022. White contours depict significant reactions and are interpreted based on the arrows. ← and → arrows show in-phase and anti-phase comovements; ↗ or ↙ arrows indicate a lead role for the first variable (GPR); ↘ or ↖ indicates a lead role of the second variable (stock returns). GPR leads (lags) stock returns by π/2 with ↑ (↓) arrows. The colour bar shows the strength of comovements – hotter (yellow to red) colours signify strong comovements and colder colours (green to blue) signify weak comovements.

Fig. 5 displays the SWC measure and the WCPD-based lead-lag dynamics between GPR and stock returns from Indonesia. For the GPR-Indonesia pair, this study documents varying coherence levels of high, medium, and low. Similar to what was observed in Fig. 2, the heatmap is mostly red above the fortnightly scale (i.e., above 16 daily cycles), depicting a high coherence during the sample period analysed. This is especially observable in the left upper quadrant, suggesting that during Russia's invasion of Ukraine in February 2022, Indonesian stock markets suffered from the Black Swan event. The consistent high coherence is evidence of the high comovements between the geopolitical risk (GPR) shocks propagated by the Russian-Ukrainian conflict and stock market returns from Indonesia. The cloud of right-upward-oriented arrows observed in this time-frequency region is an indication of mild-to-strong positive comovements, which are confirmed by the green (daily to weekly frequency band) and reddish-yellow (fortnightly to monthly frequency band) colour surrounded by the white contour in the WCPD plot (see Fig. 5-panel B). This indicates that returns on Indonesian stocks were positively driven by GPR shocks from the onset of Russia's invasion of Ukraine.

The mix of interspersed positioning arrows across 8–12 daily periodicities between mid-March and the beginning of June reveal negative comovements, as confirmed by the blue-coloured contours from the WCPD plot (see Fig. 5-panel B), suggesting possible diversification benefits. Beyond or below this frequency band, the lead-lag dynamics between GPR and Indonesian stocks leave behind no diversification benefits as the accompanying WCPD plot shows positive comovements.

### 4.5 Geopolitical risk and mexican stocks

**Fig. 6:**
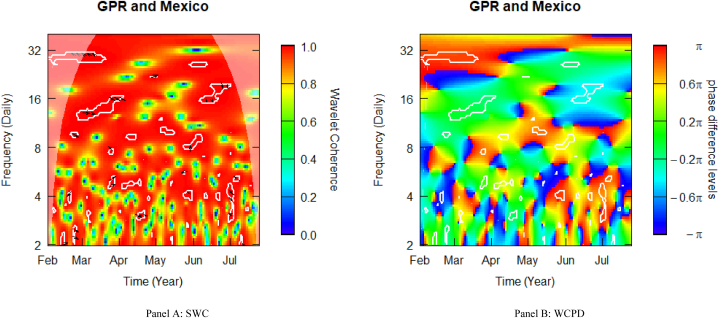
Wavelet analysis: geopolitical risk (GPR) index and stock returns in Mexico. *Notes:* This figure shows the squared wavelet coherence (left plot, i.e., Panel A) and wavelet coherence phase difference (right plot, i.e., Panel B) between GPR and stock returns in Mexico. The horizontal (vertical) axis shows time steps in months (frequency scales in days). The sample period starts from 01-Feb-2022 to 25-Jul-2022. White contours depict significant reactions and are interpreted based on the arrows. ← and → arrows show in-phase and anti-phase comovements; ↗ or ↙ arrows indicate a lead role for the first variable (GPR); ↘ or ↖ indicates a lead role of the second variable (stock returns). GPR leads (lags) stock returns by π/2 with ↑ (↓) arrows. The colour bar shows the strength of comovements – hotter (yellow to red) colours signify strong comovements and colder colours (green to blue) signify weak comovements.

Fig. 6 shows the SWC measure and the WCPD-based lead-lag dynamics between GPR and stock returns from Mexico. For the GPR-Mexico pair, this study documents varying coherence levels of high, medium, and low. Similar to what was observed in the case of Brazil (see Fig. 2), the heatmap is frequently red above the fortnight frequency band (i.e., above 16 daily cycles), depicting a high coherence during the sample period analysed. This is especially observable in the left upper quadrant, suggesting that during Russia's invasion of Ukraine in February 2022, Mexican stock markets suffered from the Black Swan event. The consistent high coherence is evidence of the high comovements between the geopolitical risk (GPR) shocks propagated by the Russian-Ukrainian conflict and stock market returns from Mexico. The cloud of right-down- (2–3 daily frequencies), and left-up- (28–32 daily cycles) oriented arrows observed in this time-frequency region is an indication of mild-to-strong positive comovements, which are confirmed by the green and reddish-yellow colour surrounded by the white contour in the WCPD plot (see Fig. 6-panel B). This indicates that returns on Indonesian stocks were positively driven by GPR shocks from the onset of Russia's invasion of Ukraine.

It is worth noting that there existed potential diversification benefits with the left-down-oriented positioning arrow found at the 4-daily frequency band in February. However, given the strength of this Black Swan period, this diversification potential was overturned at all frequency bands in the remaining periods until mid-June and early July, particularly around 14–21 daily (3-weekly) periodicities. The mix of interspersed positioning arrows across the remaining frequency bands reveals positive comovements, as confirmed by the red or green coloured contours from the WCPD plot (see Fig. 6-panel B), suggesting no possible diversification benefits.

### 4.6 Geopolitical risk and Russian stocks

**Fig. 7:**
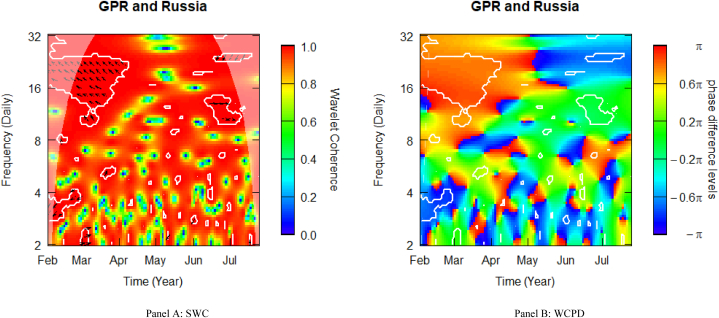
Wavelet analysis: geopolitical risk (GPR) index and stock returns in Russia. *Notes:* This figure shows the squared wavelet coherence (left plot, i.e., Panel A) and wavelet coherence phase difference (right plot, i.e., Panel B) between GPR and stock returns in Russia. The horizontal (vertical) axis shows time steps in months (frequency scales in days). The sample period starts from 01-Feb-2022 to 25-Jul-2022. White contours depict significant reactions and are interpreted based on the arrows. ← and → arrows show in-phase and anti-phase comovements; ↗ or ↙ arrows indicate a lead role for the first variable (GPR); ↘ or ↖ indicates a lead role of the second variable (stock returns). GPR leads (lags) stock returns by π/2 with ↑ (↓) arrows. The colour bar shows the strength of comovements – hotter (yellow to red) colours signify strong comovements and colder colours (green to blue) signify weak comovements.

Fig. 7 shows the SWC measure and the WCPD-based lead-lag dynamics between GPR and stock returns from Russia. For the GPR-Russia pair, this study documents time- and frequency-varying coherence levels of a high, medium, and low. Similar to what was observed in the case of other E7 stock markets, the heatmap is frequently red above the fortnight frequency band (i.e., above 16 daily cycles), depicting a high coherence during the sample period analysed. This is especially observable in the left upper quadrant, suggesting that during the invasion of Ukraine in February 2022, Russian stock markets underwent some significant changes in market dynamics. The consistent high coherence is evidence of the high comovements between the geopolitical risk (GPR) shocks propagated by the Russian-Ukrainian conflict and stock market returns from Russia. The cloud of left-up-oriented arrows observed in this time-frequency region is an indication of strong positive comovements, which are confirmed by the red colour surrounded by the white contour in the WCPD plot (see Fig. 7-panel B). This indicates that returns on Russian stocks were positively driven by GPR shocks from the onset of Russia's invasion of Ukraine. Among the E7 stock markets, Russian stocks bear the strongest positive comovements with GPR. This is attributable to the fact that these stocks originate from the initiators of the invasion. Hence, this study confirms that the degree of involvement is suggestive of the conflict's impact on stock markets, as revealed by Sun et al. [42].

From the SWC (Fig. 7-panel A), this study reveals that before the invasion, Russian stocks were rather negatively driven by GPR across the 3–4 daily frequency band, revealing potential diversification advantages. Following the invasion, the negative GPR-led comovement across high-frequency bands was overturned to a positive GPR-led comovement, thereby quashing the diversification benefits. In line with Umar, Bossman, Choi and Teplova [39] and Umar, Bossman, Choi and Vo [9], this could be attributed to the harsh economic sanctions suffered by the Russian economy from the onset of the military actions. At the apogee of the military conflict, government expenditure on defence, at the expense of other economic sectors, leads to high uncertainty and, hence, it is natural to expect that investors would act anytime they envisage a rise in the level of uncertainty. The freezing of Russia's forex reserves by the US and the imposition of detrimental sanctions on Russian citizens and businesses in late (early) February led to amplified levels of risk surrounding firms' operations, expected cash flows, profitability, and stock prices. Hence, it is comprehensible that Russian equities seem to have been more susceptible to GPR shocks, particularly across the weekly-to-monthly frequency band in the Russian-Ukrainian military conflict Black Swan. However, after some months into the sanctions, the Russian stock market seems to have made significant recoveries as there existed potential diversification benefits across the 20–24 daily cycles in June, as evidenced by the blue coloured contours from the WCPD plot (see Fig. 7-panel B), signifying some diversification benefits.

### 4.7 Geopolitical risk and Turkish stocks

**Fig. 8:**
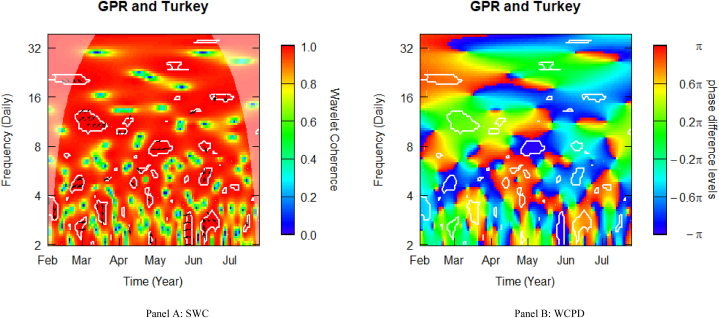
Wavelet analysis: geopolitical risk (GPR) index and stock returns in Turkey. *Notes:* This figure shows the squared wavelet coherence (left plot, i.e., Panel A) and wavelet coherence phase difference (right plot, i.e., Panel B) between GPR and stock returns in Turkey. The horizontal (vertical) axis shows time steps in months (frequency scales in days). The sample period starts from 01-Feb-2022 to 25-Jul-2022. White contours depict significant reactions and are interpreted based on the arrows. ← and → arrows show in-phase and anti-phase comovements; ↗ or ↙ arrows indicate a lead role for the first variable (GPR); ↘ or ↖ indicates a lead role of the second variable (stock returns). GPR leads (lags) stock returns by π/2 with ↑ (↓) arrows. The colour bar shows the strength of comovements – hotter (yellow to red) colours signify strong comovements and colder colours (green to blue) signify weak comovements.

Fig. 8 depicts the SWC measure and the WCPD-based lead-lag dynamics between GPR and stock returns from Turkey. For the GPR-Turkey pair, this study documents varying coherence levels of high, medium, and low. Similar to what was observed in the case of the other E7 markets, the heatmap is mostly red above the fortnightly scale (i.e., above 16 daily cycles), depicting a high coherence during the sample period analysed. This is particularly observable in the left upper quadrant, suggesting that during Russia's invasion of Ukraine in February 2022, Turkish stock markets underwent stress conditions. The consistent high coherence is evidence of the high comovements between the geopolitical risk (GPR) shocks propagated by the Russian-Ukrainian conflict and stock market returns from Turkey. The clouds of right-upward- (10–14 daily frequency band) and left-upward- (18–21 daily frequency band) oriented arrows observed in this time-frequency region are indications of mild-to-strong positive correlations, which are confirmed by the green and red colours surrounded by the white contour in the WCPD plot (see Fig. 8-panel B). However, within the same period, the cloud of left-down-oriented positioning arrows at the 4–6 daily frequency band signified diversification benefits. It is worth noting that before Russia invaded Ukraine, the diversification potential existed between 3 and 4 daily periodicities in early February.

Among the E7 stock markets, Turkish stocks reveal more instances of potential diversification in the sample period. This is evidenced by the clouds of ← and ↙ positioning arrows in blue colours surrounded by white contours found across the time-frequency space, particularly between May and July, albeit with a few interspersed red-coloured contours. The alternating position of Turkish stocks confirms the highly volatile character of EMEs during crises.

### 4.8 Summary of comovements between GPR and E7 stocks

First, GPR significantly drives the pricing- and return-generating dynamics in the E7 stock markets, as indicated by the predominant red colour of the heatmap across the fortnightly scale. This corroborates the observation of Umar et al. [9], who report that GPR significantly drives shorted stocks from various sectors of economic activity. Second, E7 stocks retain their hedging attributes in the short term, particularly during 2-weekly trading periods. Third, relative to their counterpart E7 members, Mexico and India exhibit fewer diversification attributes in the analysed period. Fourth, Russian equities made a quick recovery to revert to their diversification prospects after a few weeks into the military actions. Hence, Russian equities join their counterparts from Brazil, China, Indonesia, and Turkey to serve as safe-havens and hedges against geopolitical risk-induced shocks.

## 5 Robustness

To ascertain the robustness of the results and conclusions, the overall and net connectedness between E7 markets in the presence of geopolitical risk is examined. The time-varying parameter vector autoregression (TVP-VAR)-based connectedness metric of Antonakakis et al. [50] is employed. Through Koop and Korobilis' [51] Kalman filter technique with forgetting factors, the TVP-VAR connectedness approach allows a variation of the variance-covariance matrix. With this feature, missing observations and arbitrary specification of rolling window size, which are drawbacks of connectedness techniques like those of Diebold and Yilmaz [52] and Baruník and Křehlík [18], are avoided. Due to these advantages, the TVP-VAR connectedness approach has been employed in recent studies for either their main analysis or robustness checks [[53], [54], [55]].

The TVP-VAR spillover matrix that shows the connectedness between the E7 markets in the face of GPR is presented in Table 2. Following the existing literature, such as Akhtaruzzaman et al. [55], the results are based on a lag length of order one and a 10 step-ahead generalised forecast error variance decomposition.

**Table 2:** Average connectedness matrix.

	Brazil	China	India	Indonesia	Mexico	Russia	Turkey	GPR	FROM
Brazil	54.89	1.29	6.77	6.21	23.35	0.48	3.2	3.81	45.11
China	1.22	84.26	2.55	1.32	0.51	3.01	1.18	5.95	15.74
India	5.89	1.31	47.29	11.88	18.58	3.83	11	0.21	52.71
Indonesia	7.1	0.5	15.74	61.13	10.63	0.32	3.11	1.46	38.87
Mexico	19.34	0.7	17.79	7.38	46.27	2.16	4.99	1.37	53.73
Russia	0.5	2.6	5.76	0.15	1.91	81.58	2.23	5.26	18.42
Turkey	3.48	0.58	15.71	3.26	7.17	1.97	67.25	0.58	32.75
GPR	0.53	0.34	0.11	0.99	0.62	0.12	0.26	97.02	2.98
TO	38.06	7.33	64.45	31.19	62.78	11.88	25.98	18.64	260.31
Inc. Own	92.94	91.59	111.73	92.32	109.05	93.47	93.22	115.67	**TCI**
NET	−7.06	−8.41	11.73	−7.68	9.05	−6.53	−6.78	15.67	32.54

Notes: This table presents the average connectedness between E7 stocks and GPR over the period between July 2021 to July 2022.

**Fig. 9:**
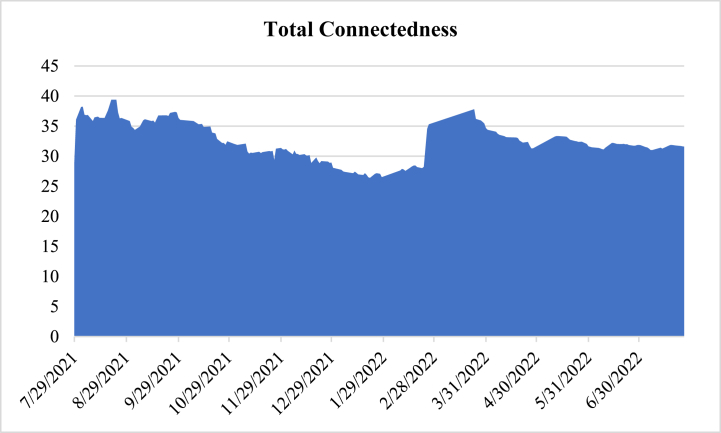
Time-varying spillover connectedness.

**Fig. 10:**
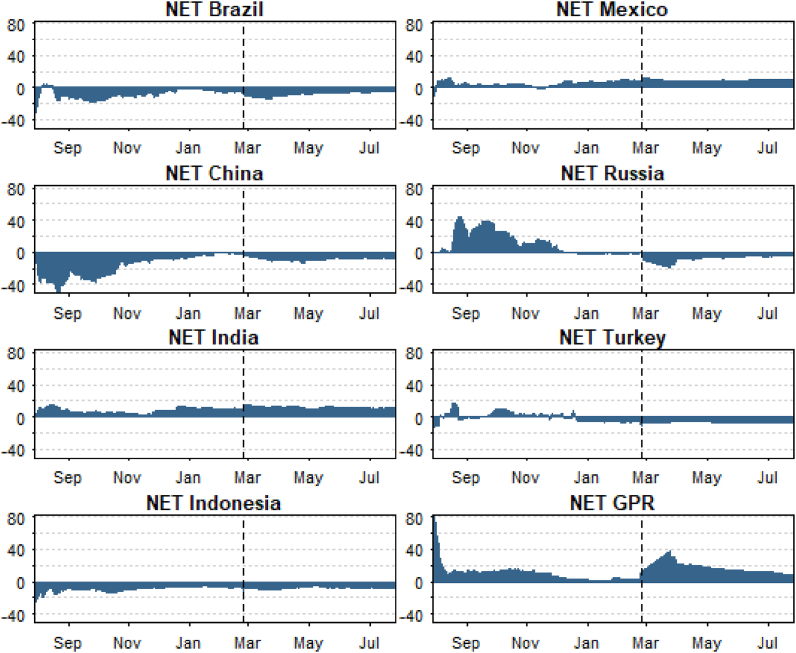
Time-varying spillover connectedness.

The results suggest that the overall connectedness between E7 markets, expressed by the total connectedness index, is 32.54%. Therefore, about a third of the variations in one of the E7 markets is explained by the connectedness between all seven markets and GPR. From the spillover matrix, the important results that need to be focused on, per the theme of the current study, are the net spillovers. The last row “NET” shows that Brazil (−7.06%), China (−8.41%), Indonesia (−7.68%), Russia (−6.53%), and Turkey (−6.78%) are all net recipients. The fact that five out of seven markets are net recipients indicates that they offer potential hedging advantages against geopolitical risk-induced shocks. Interestingly, GPR is a (and the greatest) net transmitter of system spillovers, indicating that shock transmission from other markets to E7 markets is championed by the GPR. To analyse this further, we take a look at the overall and net time-varying spillovers in Fig. 9, Fig. 10, respectively.

Fig. 9 shows the time-varying connectedness between the E7 markets and GPR. The overall connectedness between E7 markets was found to be reverting to a normal level of around 25% between December 2021 and January 2022. However, the TCI picked up a peak in February 2022 following Russia's invasion of Ukraine. This highlights the impact of GPR on E7 markets, as previously highlighted under the wavelet analysis. To ascertain the contribution of each member of the E7 markets, Fig. 10 shows that since the commencement of the military actions between Russia and Ukraine, several E7 markets (Brazil, China, Russia, Indonesia, and Turkey) are net recipients, emphasising their hedging attribute against market shocks propagated by geopolitical risk (i.e., GPR), as already discussed. Again, GPR remains a consistent transmitter of spillovers across the analysed period. Overall, the robustness of the findings from the wavelet analysis is confirmed, implying that the conclusions drawn from the study's findings are robust to different techniques.

## 6 Conclusions

This study investigates the time- and frequency-varying comovements between geopolitical risk (GPR) and the stock markets of the top-seven emerging (E7) countries. The daily GPR index developed by Caldara and Iacoviello [30] is employed in addition to the daily stock market returns of each of the E7 countries from February to July 2022. The study employs the squared wavelet coherence and wavelet phase difference techniques. The robustness of the findings is supported by the TVP-VAR connectedness approach.

The results underscore predominant medium-to-high time-frequency coherence between stock returns from E7 countries and the Russian-Ukrainian conflict-induced geopolitical risk. The largely high comovements spotted from this study imply that there exist high comovements between Black Swan events like the Russian-Ukrainian conflict and financial markets’ volatility, highlighting the essence of alternative assets or asset classes for hedging geopolitical risks in the ongoing military actions. It is worth noting, however, that the lead-lag and comovement dynamics between GPR and E7 stocks reveal some intervals of mild coherence across the time-frequency spectrum. The intervals of low coherence suggest that based on the time and frequency (i.e., trading horizon), E7 stocks create room for diversification, implying that they could be potential safe-havens during Black Swan events, like geopolitical and military conflicts. Furthermore, this study reveals that E7 stocks exhibit market-specific coherence and lead-lag patterns regarding their interdependence with geopolitical risk, communicating heterogeneous and asymmetric market responses. Hence, the findings back the use of emerging markets equities by international investors seeking diversification and downside hedging strategies. Thus, E7 stocks, particularly those from Brazil, China, Russia, Indonesia, and Turkey could be used to hedge against geopolitical risk-induced shocks. Similarly, stocks that propagate shocks during elevated periods of the ongoing geopolitical conflict could be hedged against or diversified using a blend of E7 stocks.

The results revealed from this study have notable implications for managers of portfolios, investors, and policymakers. To portfolio managers and investors, the findings guide asset allocation, particularly during Black Swan events. Given that the E7 economies have different geographical backgrounds, albeit a few shared characteristics, they could be employed for cross-regional hedging strategies by monitoring country- or market-specific attributes across different time scales as well as frequencies. In effect, developed market equities, which may be a source of contagion, could be diversified or hedged against by employing assets from emerging markets. To policymakers, the findings from this study should be instrumental in devising policies that mitigate market volatility during intense geopolitical shocks and heightened market uncertainty. Similarly, by monitoring the levels of GPR, policymakers could revise policy actions anytime GPR elevates or drops massively. This is because elevated levels of GPR could increase spillover transmission and contagion. Future works may extend this evidence by employing other econometric techniques and quantifying the portfolio implications for including E7 stocks in international portfolios.

## Author contribution statement

Samuel Kwaku Agyei: Conceived and designed the experiments; Performed the experiments; Analysed and interpreted the data; Contributed reagents, materials, analysis tools or data; Wrote the paper.

## Funding statement

This research did not receive any specific grant from funding agencies in the public, commercial, or not-for-profit sectors.

## Data availability statement

The authors do not have permission to share data.

## Declaration of interest's statement

The authors declare no competing interests.

## Footnotes

1 Black Swan was coined by Ref. [56] to describe unusual events that cause significant changes in financial markets [46]. Examples of such events are financial crises, wars, health crises, terror attacks, natural disasters, and elections.
